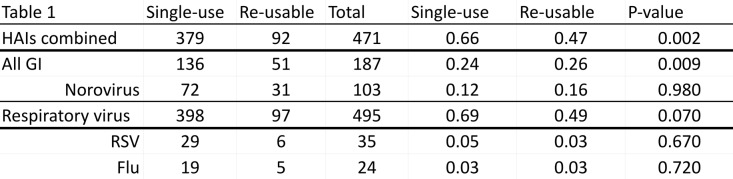# 352 Exploring the Relationship Between Hysterectomy Surgical Site Infection and Area Deprivation Index

**DOI:** 10.1017/ash.2026.10691

**Published:** 2026-06-23

**Authors:** Prakhya Koya, Xiaoyan Song, Monica Monteon, Michelle Liberty, Annette Lee, Emma Esders

**Affiliations:** 1 Children’s National Hospital

## Abstract

**Background:** Isolation gowns are one of the most used personal protective equipment worn by healthcare workers to prevent healthcare associated infections (HAIs) that can spread via direct- or indirect contact of contaminated objects. Isolation gowns are available as either reusable or disposable; however, urgent data are needed to determine whether these two gown types are associated with differences in HAI rates. Objective: To assess if respiratory and gastrointestinal viral HAI rates differ by using disposable versus reusable gowns. Methods/design: A retrospective observational analysis compared HAI rates per 1000 patient-days before and after phased implementation of reusable gowns across inpatient units from July 2020 through November 2025. to examine the significance of differences in HAI rates. Results/discussion: The analysis identified 513 HAIs, including 314 respiratory and 169 gastrointestinal viral infections. Statistically significant lower HAI rates were observed with reusable gowns compared with disposable gowns for all HAIs combined (0.75 vs. 1.07, p=0.002) and gastrointestinal HAIs (0.26 vs. 0.35, p=0.009). Lower but statistically insignificant rates were observed for respiratory viral HAIs (0.49 vs. 0.72, p=0.07). Rates were comparable or lower with reusable gown use for norovirus (0.16 vs. 0.16, p=0.98), respiratory syncytial virus (0.03 vs. 0.04, p=0.67), and influenza (0.03 vs. 0.02, p=0.72). Implementation of reusable gowns was not associated with increased respiratory HAI rates and was associated with lower overall HAI incidence. These findings suggest that reusable gowns provide effective protection against respiratory pathogen transmission while supporting sustainability initiatives. Reusable gowns may represent a viable strategy to balance infection prevention, environmental impact, and resource utilization in healthcare settings.

**Figure:**
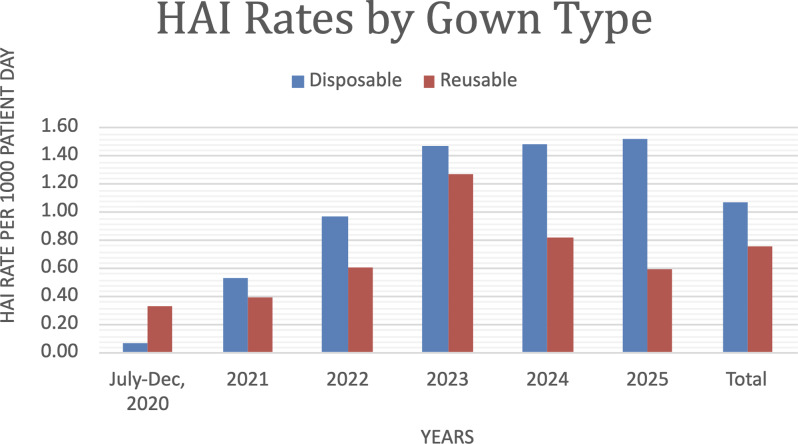

**Figure:**